# Highly Sensitive Automated Method for DNA Damage Assessment: Gamma-H2AX Foci Counting and Cell Cycle Sorting

**DOI:** 10.3390/ijms140815810

**Published:** 2013-07-30

**Authors:** Laia Hernández, Mariona Terradas, Marta Martín, Laura Tusell, Anna Genescà

**Affiliations:** Department of Cell Biology, Physiology and Immunology, Universitat Autònoma de Barcelona, 08193 Bellaterra, Spain; E-Mails: laia.hernandez@uab.es (L.H.); mariona.terradas@uab.es (M.T.); marta.martin@uab.es (M.M.); laura.tusell@uab.es (L.T.)

**Keywords:** gamma-H2AX, cell cycle, automated screening method, dosimetry, DNA damage, mammography

## Abstract

Phosphorylation of the H2AX protein is an early step in the double strand break (DSB) repair pathway; therefore, phosphorylated histone (γH2AX) foci scoring is widely used as a measure for DSBs. Foci scoring is performed either manually or semi-automatically using hand-operated capturing and image analysis software. In general, both techniques are laborious and prone to artifacts associated with manual scoring. While a few fully automated methods have been described in the literature, none of them have been used to quantify γH2AX foci in combination with a cell cycle phase analysis. Adding this feature to a rapid automated γH2AX foci quantification method would reduce the scoring uncertainty that arises from the variations in the background level of the γH2AX signal throughout the cell cycle. The method was set up to measure DNA damage induced in human mammary epithelial cells by irradiation under a mammogram device. We adapted a FISH (fluorescent in situ hybridization) Spot-counting system, which has a slide loader with automatic scanning and cell capture system throughout the thickness of each cell (z-stack), to meet our assay requirements. While scanning the sample, the system classifies the selected nuclei according to the signal patterns previously described by the user. For our purposes, a double staining immunofluorescence was carried out with antibodies to detect γH2AX and pericentrin, an integral component of the centrosome. We could thus distinguish both the number of γH2AX foci per cell and the cell cycle phase. Furthermore, restrictive settings of the program classifier reduced the “touching nuclei” problem described in other image analysis software. The automated scoring was faster than and as sensitive as its manually performed counterpart. This system is a reliable tool for γH2AX radio-induced foci counting and provides essential information about the cell cycle stage. It thus offers a more complete and rapid assessment of DNA damage.

## 1. Introduction

The need to assess DNA damage after exposure to ionizing radiation has prompted research into finding specific molecular markers for DNA lesions and the DNA damage response. The study of histone H2AX phosphorylation has become a widely used method for DNA damage detection and the analysis of repair [1,2]. H2AX phosphorylation (γH2AX) takes place at the double strand break (DSB) site shortly after its formation, disappears at its resolution and is microscopically visible as discrete foci after antibody labeling [3,4]. As DSBs are the most toxic lesions following ionizing radiation, the proportion of γH2AX foci remaining after a certain time period has been used as a measure of the radiation sensitivity and repair efficiency of exposed cells [5–7].

Quantification of γH2AX foci can be performed either manually, by individually counting the number of foci present in each cell nucleus or automatically, by evaluating the total γH2AX immunofluorescence intensity emission per cell, using high throughput techniques, such as flow cytometry. The first method is time-consuming and is subject to the interpretation of the investigator. The second approach is more efficient, but less sensitive, and therefore, it is not suitable for low dose radiation damage assessment. To improve these methodologies, several intelligent computer pattern recognition algorithms have been developed [8,9]. These algorithms permit focus counting, as well as foci size definition. The programs process the images after capturing them, either manually or automatically. Open source programs, such as FociCounter and CellProfiler, are used to analyze images after their capture [10,11]. CellProfiler is faster than FociCounter, because it does not require treating each image and cell nuclei individually and it provides more information about the γH2AX foci and the cell nuclei. However, these methods still have some problems to overcome, such as counting “touching nuclei” or requiring large amounts of time for image exportation and processing. Other authors have reported the development of methodologies to couple motorized image capture to software, which analyses the images with pattern recognition algorithms [9]. A common feature of the works found in the literature that use these programs is that they limit their study to the number of γH2AX foci, excluding qualitative factors, such as the variations in the γH2AX foci pattern during the cell cycle.

Replicating cells (S-phase) with undamaged DNA display γH2AX constitutively [12,13]. Similarly, since the number of foci in the G2-phase are higher than in the G1-phase, this could significantly enlarge the foci-count error bars [14]. These differences have been corrected by means of flow cytometry [15,16], but no automated fluorescent microscopy system has addressed them. In this regard, we endeavored to improve the damage assessment process by incorporating a cell cycle marker to the γH2AX immunofluorescence. Our system, adapted from a FISH Spot-counting system, has a slide loader with an automatic scanning and cell capture system. Coupling these two steps is faster than processing the images after their capture. In contrast to other systems, Spot-counting immediately classifies the selected nuclei according to the signal patterns previously described by the user. In terms of pattern definition, co-staining with γH2AX and pericentrin permits considering the number of foci scored variations depending on the phase of the cell cycle. This approach also minimizes the problem of touching nuclei, which unequivocally leads to over-scoring the number of foci per cell. This problem decreases the method sensitivity, either by masking or overstating the differences among the samples. Furthermore, as it is a “one step process” where capture and classification are coupled, it is faster than current methods. We performed several experiments to set up and evaluate the sensitivity and reliability of our innovative method, with the specific purpose of assessing DNA damage induction in human mammary epithelial cells (HMECs) after irradiation with the mammogram device. This scenario required a highly sensitive DNA damage detection method, since radiation doses were very low; in fact, the minimum dose delivered to the cells in our study is equivalent to the dose received during a single mammogram exploration.

## 2. Results and Discussion

### 2.1. Improved Automated Scoring Classifies Cells According to the Cell Cycle Phase

**Figure 1:**
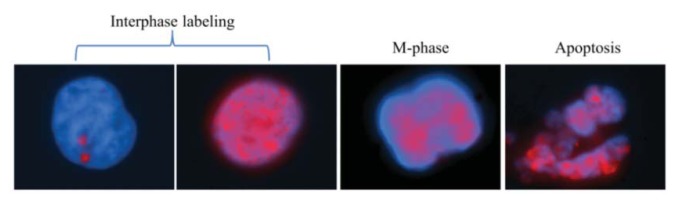
Representative images of different phosphorylated histone (γH2AX) labeling (red) patterns in combination with nuclear staining (blue) show remarkable variance in the γH2AX immunostaining patterns, not just during apoptosis, but between the different cell cycle phases on human mammary epithelial cells (HMECs).

An important consideration in γH2AX immunostaining is that the number of foci scored and the labeling pattern varies according to the phase of the cell cycle, since the histone content doubles during the cell cycle along with the doubling of DNA content [12,14]. Indeed, we observed different patterns in γH2AX immunostaining of human mammary epithelial cell lines, due to both cell cycle transition and apoptosis (Figure 1). We thus considered it essential to take such changes into account to accurately use γH2AX foci scoring as a surrogate of DNA damage induction. Using cell cycle markers and nuclear area measurements, cell cycle sorting can be performed, thereby allowing a more precise scoring of γH2AX foci in each cell cycle phase.

#### 2.1.1. Characteristic γH2AX Staining Patterns of S- and M-Phase Cells

H2AX is not only phosphorylated in response to DNA damage, but also during normal replication and in response to replication stress [17]. Thus, when γH2AX is scored as an indicator of DSBs, it is important to discriminate the radio-induced foci from those seen in S-phase cells in the absence of DNA-damage-inducing treatments.

**Figure 2:**
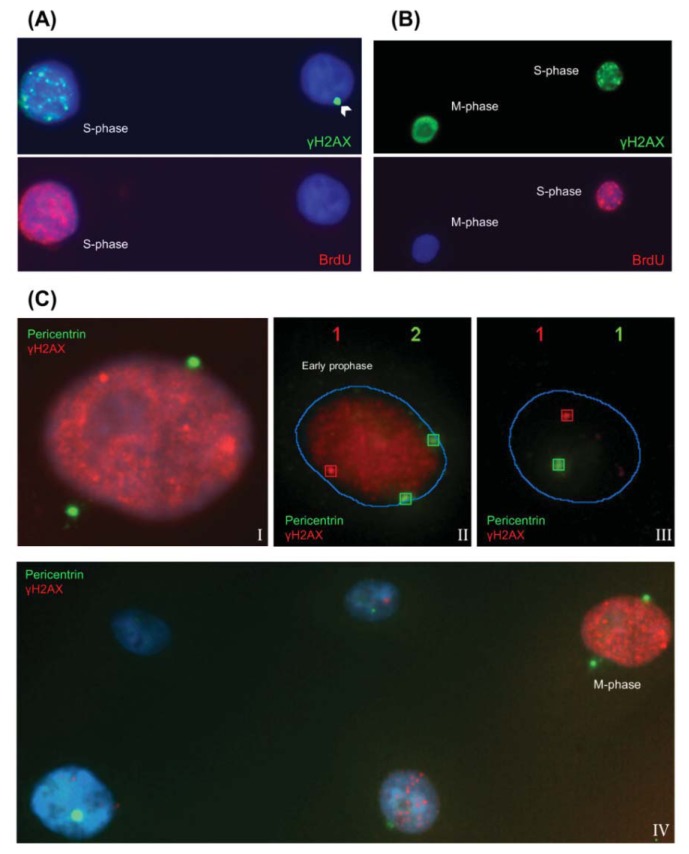
S- and M-phase γH2AX labeling pattern characterization in non-irradiated HMECs. (**A**) The top figure shows green γH2AX labeling, and the lower figure shows red bromodeoxyuridine (BrdU) labeling. S-phase nucleus (on the left) displays a characteristic γH2AX labeling pattern: a rough staining across the nucleus. None of the cell nuclei with discrete γH2AX foci (on the right) presented any BrdU labeling; (**B**) The top figure shows green γH2AX labeling, and the lower figure shows red BrdU labeling. M-phase cells (nucleus on the left), negative for BrdU, exhibit a bright pan-nuclear γH2AX labeling pattern different from the characteristic S-phase pattern (nucleus on the right) defined by both BrdU and γH2AX staining; (**C**) Pericentrin has been labeled with a green fluorochrome; two pericentrin dots can be observed during the M-phase. They are detected unengaged in early prophase stages (II) and in both poles from late prophase (I) up to the end of the mitosis. Note that at mitosis (I, II and IV), cells present a homogeneous and bright pan nuclear γH2AX staining with respect to their interphase cells counterparts (III and IV). In the images, C.II and C.III, red boxes signal γH2AX foci detected by the Spot-counting system, and green boxes signal the detected pericentrin. Boxes are drawn by the system.

To characterize the γH2AX pattern displayed by S-phase cells, we incubated human mammary epithelial cells actively growing *in vitro* (70% confluence) with the thymidine analog, bromodeoxyuridine (BrdU), which is incorporated into newly synthesized DNA strands. By means of double immunodetection of BrdU and γH2AX, we established that after a 30 min BrdU pulse in proliferating cells, 24% were positive for BrdU. The BrdU positive cells showed a characteristic granulated and rough γH2AX labeling across the nuclei, as shown in Figure 2A. The BrdU staining pattern coincided to a greater or lesser extent with the γH2AX pattern, due to their common presence in the replication forks. This γH2AX pattern can be easily distinguished from the pattern exhibited by M-phase cells. The M-phase cells’ γH2AX pattern is also pan-nuclear, but brighter, and the nuclei appear more uniformly stained (Figure 2B). Furthermore, the γH2AX labeling pattern of M-phase cells was unequivocally characterized by combining the detection of the phosphorylated histone and pericentrin, a conserved centrosome protein that is located in each spindle pole in mitotic cells (Figure 2C).

#### 2.1.2. CENP-F to Identify G2 Cells and Define the Nuclear Area Range of Cells in G2

**Figure 3:**
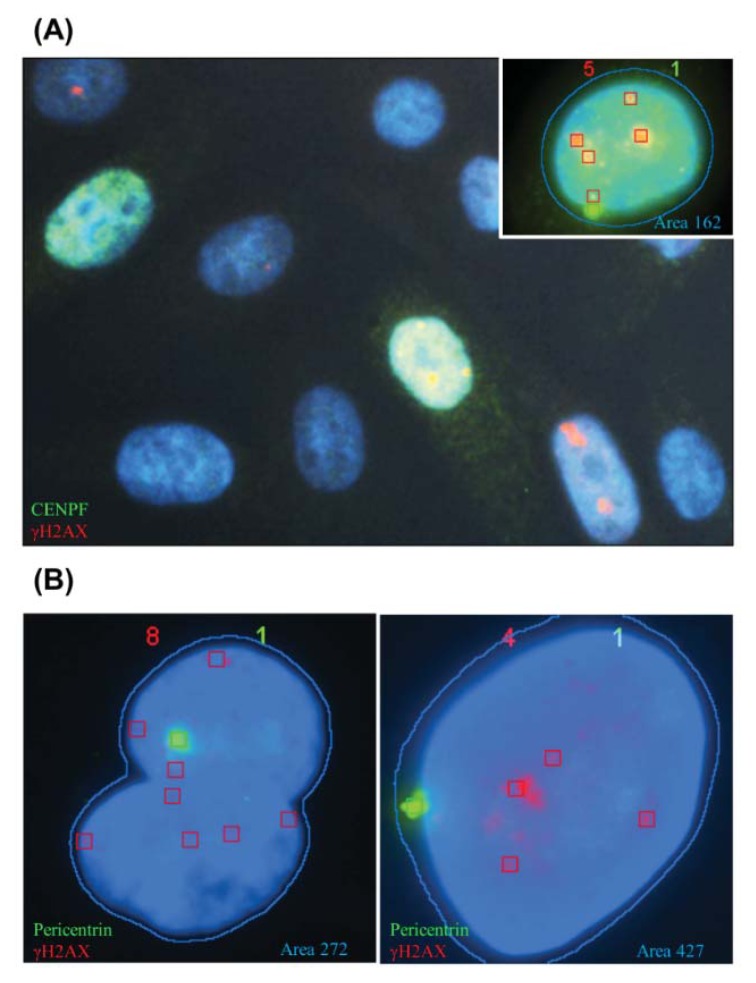
Centromere protein F (CENP-F) staining used for defining G2 cell area range. (**A**) CENP-F, which accumulates to become abundant in G2, was found in cells from 130 to 250 nuclear area points, with a mean value of 162.7 points for CENP-F positive cells; (**B**) nuclear area measurements above 250 probably corresponded to overlapped nuclei or polyploidy cells. Red boxes signal γH2AX foci detected by the system. Boxes are drawn by the system.

With the aim of discriminating G2 from G1 cells, we analyzed the presence of centromere protein F (CENP-F), a kinetochore protein that gradually accumulates in G2- and M-phase cells. The analysis of the CENP-F expression was performed in growing HMEC-hTERT cells. By direct observation under an epifluorescence microscope, we established that 14% of cells were CENP-F positive (Figure 3A, 200 cells analyzed). As the Spot-counting system cannot analyze both CENP-F and γH2AX, we decided to use the nuclear area as a surrogate of CENP-F staining in order to distinguish cells in G2 from G1 cells. To estimate which was the nuclear area range of CENP-F positive cells, we used the automatic capture and analysis mode of the Spot-counting system (Figure 3A). We performed two consecutive rounds of staining; first, a γH2AX and pericentrin co-staining and, secondly, γH2AX and CENP-F. Using the re-localization function of the Spot-counting system, we restricted our nuclear area analysis to CENP-F positive cells with only one pericentrin foci, disregarding any cell in the M-phase. We determined that the mean area for CENP-F positive cells was 162.7 points, with a range between 130 and 250. We couldn’t find any CENP-F positive cells over 266 points of cell nuclei surface size, nor any less than 130 pts. Furthermore, we observed that cellular nuclei under 50 area points were commonly debris, and cells displaying a nuclei area over 250 points were likely to be either overlapped nuclei or polyploid cells (Figure 3B). We therefore defined two different cell groups: 50–130 for G1 cells and 130–250 for cells in G2.

#### 2.1.3. Pericentrin: The Co-Staining Marker of Choice

**Figure 4:**
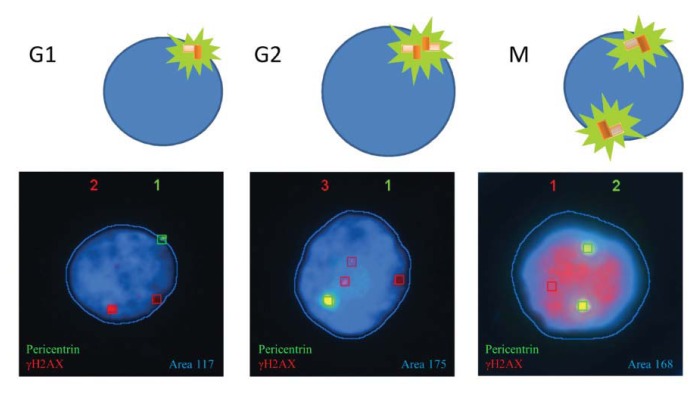
Pericentrin signal variations during the cell cycle. Surrounding the centrioles, the pericentriolar material (PCM) provides a platform for emerging microtubules. In parallel with the cell cycle progression runs, a process known as centrosome maturation, where the PCM increases in size (mid-diagram and nucleus image) and finally splits (right diagram and nucleus image) to organize a bipolar mitotic spindle necessary for chromosome segregation between daughter nuclei. Red boxes mark γH2AX foci detected by the system and green boxes mark the detected pericentrin. Boxes are drawn by the system.

Spot-counting, our system for automatic γH2AX foci scoring, was originally established for automated scoring of FISH signals. It was not prepared to count cells with no signal; thus, initially, it disregarded the cells with zero γH2AX foci. To overcome this problem, we needed to choose a constitutive marker whose expression was independent from radiation exposure. Pericentrin was our marker of choice, because of its constitutive presence in the cell, frequently overlapping the nuclear area. Pericentrin, as described before, is a conserved protein of the pericentriolar material (PCM) [18], which surrounds the centrioles and plays an important role in microtubule organization and, as illustrated in Figure 4, varies in size and distribution along the cell cycle. Besides allowing us to score nuclei with no γH2AX foci, pericentrin labeling also allowed distinguishing late G-2 and M-phase cells, when both centrosomes are unengaged. Therefore, pericentrin was chosen as the most suitable co-staining marker in combination with γH2AX.

**Figure 5:**
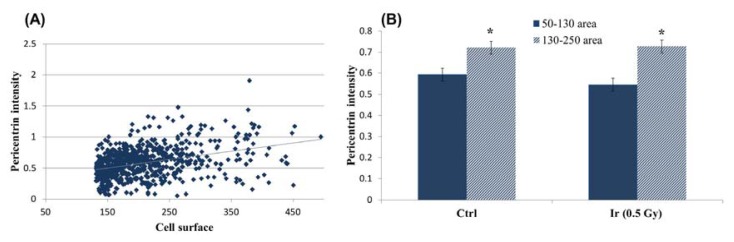
Nuclear area *vs*. pericentrin signal intensity. (**A**) Plotting cell surface measurements *versus* pericentrin intensity shows a positive correlation between the two variables: pericentrin intensity increases as the area increases. 1000 unsorted cells were plotted; (**B**) When sorting cells by area range, the mean pericentrin intensity value is found significantly higher in the 130–250 area groups, as compared to the 50–130 area groups. A single asterisk (*) refers to statistically highly significant difference (*p* < 0.0001). One-thousand cells per dose were analyzed 2 h post irradiation and two replicates per time point.

In addition, pericentrin labeling could also help discriminate G1 from G2 cells, when combined with automatic cell surface measurements. The Spot-counting system is able to count the number and measure the intensity of each dot captured. Using the information of pericentrin signal intensity, we plotted this intensity *versus* the area stained with 4′,6-diamidino-2-phenylindole (DAPI) (Figure 5A). A good correlation was found between pericentrin intensity and nuclear area, especially when the cells were growing exponentially (Spearman *r* = 0.4942, *p* < 0.0001). The experiment was performed using cells from three different donors (for further information, see the Experimental Section). The correlation coefficient ranged from 0.34 to 0.6 for the different donors when only one pericentrin signal was observed, all the correlations being extremely significant (*p* < 0.0001). As expected, when cells were reclassified by the defined area groups, the pericentrin signal was significantly brighter in the 130–250 area group, both in irradiated and in non-irradiated cell samples *(p* < 0.0001, Mann Whitney test) (Figure 5B). In addition, the percentage of cells in the big area group (16%, Donor 1, 70% confluence) coincided with the percentage of CENP-F positive cells (14%, Donor 1, 70% confluence).

These positive correlations encourage the use of nuclear area measurements as a G1/G2 classifier, as it clearly corresponds to the changes that the cell undergoes along the cell cycle. During its progression through the cycle, the cell coordinately replicates the DNA material and duplicates the centrosome material, which, at the end of G2, is split in order to organize an effective bipolar mitotic spindle. The nuclei surface measurement classification reinforced by the pericentrin intensity data is a double check for the cell cycle sorting, providing a consistent γH2AX scoring on sorted cells. The accuracy of the area group classification and the use of pericentrin as a strategy to gather additional cell information are evidence of the system reliability.

### 2.2. γH2AX Automatic Foci Counting

#### 2.2.1. Foci Analysis

The foci analysis is performed rapidly, though the time consumed depends on the culture confluence and whether the sample has been irradiated or not. We calculated that it takes 3 h from setting up the microscope platform to obtain the data and store it in a spreadsheet for a 0.5 Gy irradiated sample (70% confluence, 2 h post-irradiation). This time would be reduced in a control sample. The information given by the system is more extended than that obtained by manually counting, as it provides data on the foci intensity (minimum and maximum) and the cell nuclei diameter in addition to the number of foci per cell. To evaluate whether the system was able to perform a consistent scoring, four samples (two controls and two 0.5 Gy irradiated) were scored twice. The differences in the mean foci count were barely detectable; none of the samples displayed a difference higher than 0.05 foci/cell within the two counting rounds. When working with replicates (two irradiated with 0.5 Gy samples and two irradiated with 1 Gy samples and their respective controls), the differences between their mean foci counts (in non-sorted cells) vary from a minimum of 0.06 foci/cell to 0.56 foci/cell. The smallest differences were detected between the controls (1.12 ± 0.06), and they became greater with radiation dose: 0.5 Gy (4.75 ± 0.36) and1 Gy (5.23 ± 0.56). Thus, when the immunostaining of the sample is adequate and the capture conditions are steady, the Spot-counting system homogenously and repeatedly gave very similar scoring results.

#### 2.2.2. γH2AX Signal Saturation at High Radiation Doses in Non-Sorted Cells

**Figure 6:**
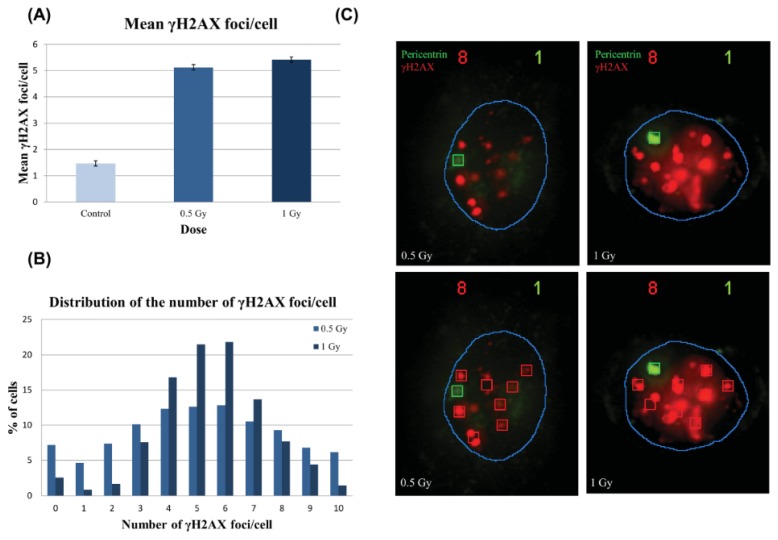
γH2AX radio-induced foci number, distribution and size. (**A**) The average number of foci per cell was statistically significantly higher (*p* < 0.0001, Mann Whitney test) in both 0.5 and 1 Gy samples with respect to their controls, but the increase between 0.5 and 1 Gy, despite being statistically significant (*p* < 0.01, Mann Whitney test), was smaller than expected; (**B**) The foci distribution was tighter in the 1 Gy sample than in the 0.5 Gy sample, where the distribution was broader; (**C**) Representative images to illustrate the observed increase in the size of radio-induced foci with dose. As a result, 1 Gy γH2AX foci signals are wider than 0.5 Gy foci. One-thousand cells per dose were analyzed 2 h post-irradiation and two replicates per time point. Red boxes signal γH2AX foci detected by the system, and green boxes signal the detected pericentrin. The blue line is drawn by the system and delimitates the nucleus.

To evaluate the use of the Spot-counting method for DNA damage induction assessment, we irradiated Donor 1 HMECs, previously immortalized by hTERT gene transduction, with 0.5 and 1 Gy. When plotting the dose *versus* the mean number of γH2AX foci per cell scored in Donor 1 cells (not sorted by area), we found that the average number of foci per cell increased with the irradiation dose. However, the increase exhibited by the cells irradiated with 1 Gy was smaller than expected (Figure 6A). This observation could be due to a saturation effect caused by overlapping foci signals in the cells irradiated at the highest dose. To unravel this problem, we analyzed the number of foci per cell (Figure 6B). We observed that the distribution of foci per cell in the 1 Gy sample was narrower than in the 0.5 Gy sample: less cells with zero foci, as was expected for cells irradiated with a higher dose, but also a diminished number of cells with 10 foci. We hypothesized that the cells containing more than five foci in the 1 Gy sample are not being properly classified, because they are not seen as discrete foci, since they overlap, forming larger, but fewer, foci (Figure 6C).

**Figure 7:**
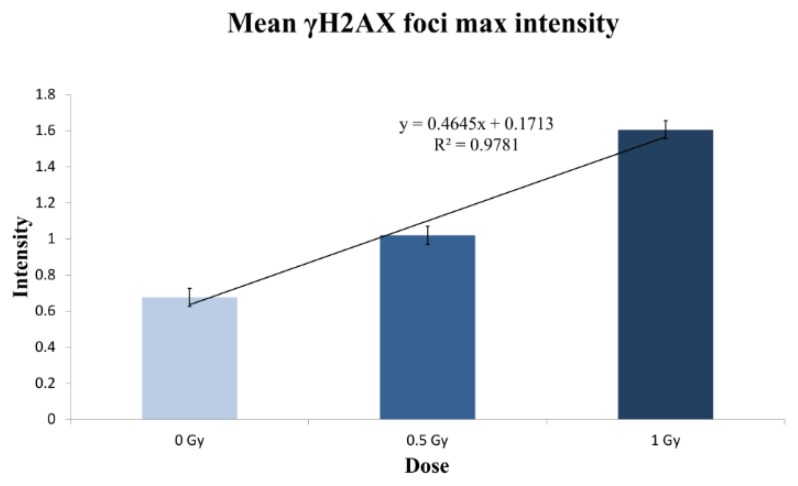
The mean γH2AX foci intensity increases linearly with radiation dose. Two-thousand unsorted cells per dose were analyzed 2 h post-irradiation. Note the high value of the coefficient of determination, *r*^2^ = 0.9781.

We wanted to check if the qualitative perception of the foci becoming wider with increasing dose was measurable. With that purpose, we used the mean intensity value of the brightest foci of each cell, information also provided by the Spot-counting system. As seen in Figure 7, the mean γH2AX foci max intensity increases strongly, depending on the dose (*r*^2^ = 0.9781). This result supports the idea that the overlap between the foci is more pronounced among higher doses, corresponding to the moderate difference observed between the mean number of foci in the 0.5 Gy sample and the 1 Gy sample. We can envisage two reasons to explain why higher doses of radiation lead to larger foci: the foci signals become wider with the dose and, as the number of radio-induced DSBs rises, thus the chance of two foci placed together and scored as one is greater.

**Figure 8:**
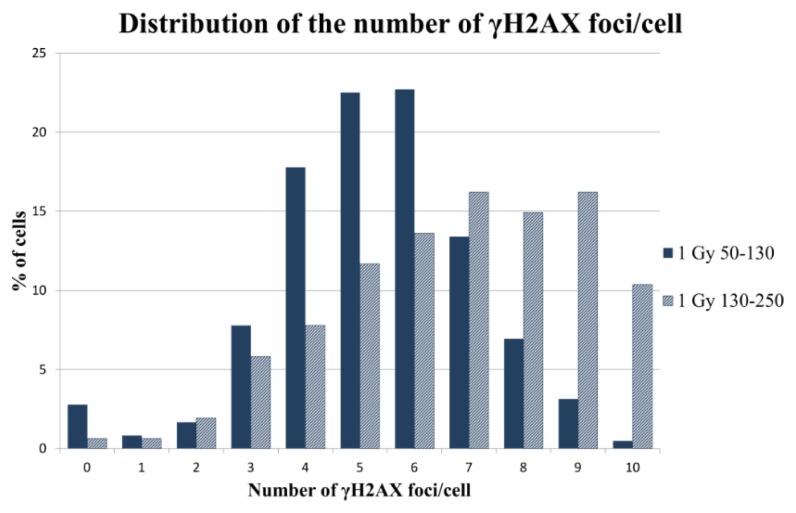
The foci distribution is shifted to the right in G2 cells. The cells under the 130–250 range classification presented higher percentages of cell containing more than five foci than the cell classified under the small area range, *i.e.*, in G1. One-thousand sorted cells were analyzed.

In order to check whether automatic scoring of γH2AX foci in sorted cells by cell cycle phase could overcome the problems derived from signal overlapping, we scored radiation-induced γH2AX foci in cells sorted by area. The cells classified under the G2 nuclear area range displayed a significantly increased number of foci with respect to their G1 range counterparts in all the samples. As shown in the 1 Gy sample, the foci distribution of G2 nuclear area range cells was clearly shifted to the right, with higher numbers of foci per cell (Figure 8). Therefore, by means of sorting cells by area, we can limit the γH2AX foci analysis to cells in G1, in order to reduce the signal saturation effect at high doses.

#### 2.2.3. Two Mammography Shots Induce a Significant and Detectable Increase in the Number of Foci

**Figure 9:**
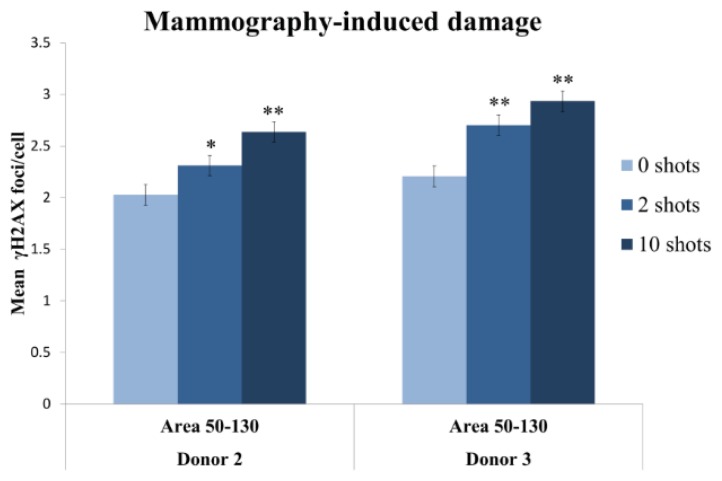
Detectable increase in the mean incidence of mammography induced γH2AX foci per cell 2 h post-irradiation in cells exposed to an X-ray dose equivalent to a single mammogram exploration. One-thousand sorted cells by area were analyzed per dose and donor. Columns represent the mean, and error bars signify standard error. An asterisk denotes a statistically significant difference between the irradiated samples and their sham-irradiated controls (Mann Whitney test). A single asterisk (*) refers to statistically significant difference (*p* < 0.01), while a double asterisk denotes a highly significant difference (*p* < 0.0001).

Our aim was to develop a method that performed rapid and automatic detection of radio-induced damage, even at very low doses. Therefore, the method had to be very sensitive. Otherwise, the radiation effects in the cells would remain unnoticed. To evaluate the sensitivity of the Spot-counting system, we irradiated vHMECs from two donors with zero, two and 10 automatic X-ray shots under a mammogram device. One single shot is equivalent to 0.01 Gy; thus, the doses received were 0.02 Gy for the cells irradiated with two shots and 0.1 Gy for the samples that received ten shots. After irradiation, we incubate them for 2 h, and a double immunostaining with pericentrin and γH2AX was performed. For the automatic scoring, we maintained the same capture settings in all experiments performed. All the data was transferred into a spreadsheet, where we delimited the area and the number of pericentrin signals, to meet all the requirements for a reliable result. The Spot-counting system was able to detect differences between controls and 10 shots, and most importantly, it was able to distinguish an increase in the mean number of γH2AX foci in the cell samples that received only two automatic X-ray shots with respect to the non-irradiated cell samples (Donor 2 and Donor 3 cells) (Figure 9). This proves that the method is highly sensitive, as two shots are equivalent to a dose received in each single standard mammogram X-ray exploration (standard entrance skin dose is 0.02 Gy per two-view mammogram exposure).

#### 2.2.4. Manual *vs.* Automatic γH2AX Foci Scoring: A Reliable Automated Approach

**Figure 10:**
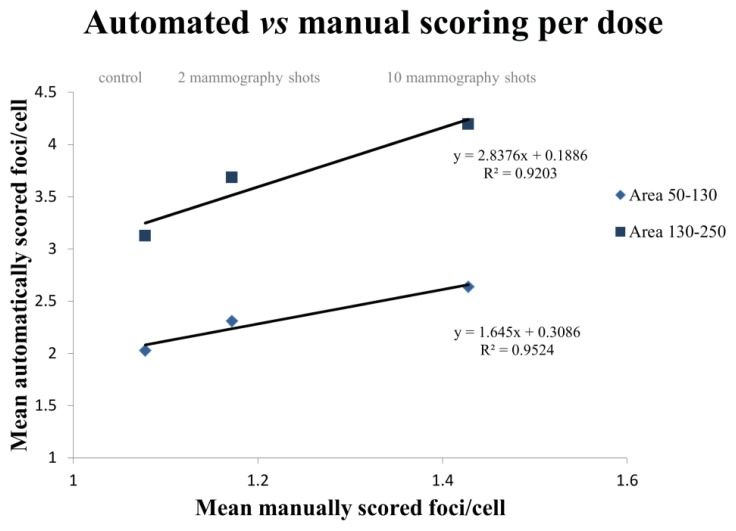
Agreement between automatic and manual scoring. One thousand cells were scored both manually and automatically for each dose (control, two mammography shots and 10 mammography shots). The mean number of foci per cell obtained with the Spot-counting system was plotted *versus* the manual scoring. The lines represent the results obtained when sorting the cells by area: 50–130 area range (rhombs) and 130–250 (squares). The small area range gave a better fitted result.

In order to evaluate if the method was reliable, we proceeded to compare automatically scored samples with their manually scored counterparts. The manually scoring was done blindly without any other labeling than γH2AX and DAPI as a counterstain. The automatic scoring was performed on the same samples used for the manual scoring with identical capture settings for all the samples, as described in the Experimental Section. Figure 10 illustrates that there was a good agreement between automated and manual scoring in all samples. However, when comparing sorted cells, the small area subpopulation gave a better fit (*y* = 1.645*x* + 0.3086, *r*^2^ = 0.9524) than big area cells (*y* = 2.8376*x* + 0.1886, *r*^2^ = 0.9203). As described before, G2 cells present a higher number of foci, so when comparing their foci values to those scored manually, where the scorer does not distinguish between phases, it is expected that the slope of the latter should be substantially greater than one.

## 3. Discussion

After defining the γH2AX labeling pattern of HMEC cells along the cell cycle, we used it in combination with cell cycle markers and area measurements to improve the accuracy of the automated scoring of γH2AX foci. The steps that we followed to define the changing labeling pattern of γH2AX and the establishment of the area measurements could be followed by any researcher to set up not just the Spot-counting system, but any automated platform. These steps provided the basis to properly sort the cells according to the cell cycle phase via our Spot-counting system, which almost completely avoids the problem of “touching nuclei” reported by other automated system users [11]. Our system is able to immediately discriminate the cells containing two pericentrin foci, thus reducing the possibility of scoring two cells as one. In addition, we studied in depth the area measurements in order to assure that the possibility of scoring two cells instead of one was ruled out. Although there is no clear specification of the time that other automated systems need for γH2AX foci scoring, using the Spot-counting system on irradiated cells (0.5 Gy) at 70% confluence, less than three hours are needed to scan, if necessary, review and export the data of 1000 nuclei into a spreadsheet. Thus, this method is definitely faster than manual scoring and any other method requiring the user’s intervention at some point (namely, image exportation or nuclei delimitation by hand).

In terms of scoring accuracy, the system tends to slightly over-score the mean number of foci, due to the small portion of zero γH2AX foci cells in which the pericentrin signal does not overlap with the nuclei and remains unseen by the system. Spot-counting results are in excellent agreement with manual scoring, demonstrating its high reliability. Nevertheless, this consistency with manual counting could be lost at high radiation dose exposures, where overlapping foci impede proper scoring. As the radiation dose increases, the number of DSBs increases, and thus, for the number of γH2AX foci, too, accordingly, the chance of overlapping is greater. For this reason, we observed a better correlation between mean foci intensity and dose than between dose and the mean number of foci. However, this is not only due to overlapping; as other authors have described, there is also an increment in the foci size with the radiation dose [8]. This loss of linearity on the dose-response curve due to overlapping could be corrected to some extent by excluding the cells in the big area range, as they are mainly cells in G2 with higher numbers of foci per cell.

Finally, the method has been proven to be highly sensitive, as it is capable of distinguishing an increase in dose at as low as 0.02 Gy, the typical radiation dose delivered at the skin level by a mammogram device. The Spot-counting system is able to measure small differences in the number of γH2AX foci, since high numbers of nuclei can be scored, and the system provides the possibility to discriminate between cell cycle phases. This discrimination allows the user to monitor the culture progression and to differentiate increments in the number of foci caused by the changes in the cell cycle from the radio-induced foci. Excluding the variations due to the cell cycle makes the system not just more sensitive, but more reliable.

## 4. Experimental Section

### 4.1. Cell Cultures

Primary non-transformed human mammary epithelial cells (HMEC) were derived from normal breast tissue of three Caucasian women. Cells were purchased from Cell Application (Donor 1, age 30), Cambrex Biowhittacker (Donor 2, age 58) and Gibco (Donor 3, age 28). The cells were seeded into cover slips or chamber slides and were grown in serum-free MepiCGS (ScienCell, research laboratories) supplemented with penicillin and streptomycin at 37 °C and 5% CO_2_. To reconstitute telomere length, HMECs from Donor 1 were transduced with viral particles containing LV.hTERT, a lentivirus construct provided by the Viral Vector Facility (CNIC, Spain), in the presence of 4 μg/mL polybrene (Sigma-Aldrich, St. Louis, MO, USA). After 24 h post-transduction, new medium was added and cells were incubated at 37 °C and 5% CO_2_. The presence of hTERT, necessary for telomerase activity, was confirmed by Western blot immunodetection (data not shown).

### 4.2. Irradiation

HMECs were irradiated with different doses: two and ten automatic shot X-rays under a mammogram X-ray diagnostic device (SENO DMR plus, General electric). Each shot was equivalent to 0.01 Gy at the skin surface (measured with an R-100 detector), which implies a 0.0027 Gy glandular dose. The X-ray molybdenum-anode tube voltage was 28 KeV, and a dose rate of 0.67 Gy/min was used. HMECs were exposed in a chamber slide support, placed on top of several methacrylate plates at a height corresponding to the average thickness of breast under compression. For experiments in Section 2.2.2, HMECs were irradiated at different doses (0.5 Gy or 1 Gy) by exposing them to ^137^Cs γ-rays at a dose rate of 5.45 Gy/min using an IBL 437C source.

### 4.3. Immunostaining

The HMECs were seeded in cover slips and grown until 70% confluence was reached. Cells were fixed (paraformaldehyde 4% for 10 min) and, then, permeabilized for 20 min in 1× PBS 0.5% TritonX100 solution, rinsed twice with 1× PBS and, then, incubated in PBS 0.1% Tween20 2% fetal calf serum for 1 h at room temperature. γH2AX, Pericentrin and CENP-F proteins were detected using mouse anti-γH2AX (Ser139) (Upstate), rabbit anti-Pericentrin (Abcam) and rabbit anti-CENPF (Abcam) at a final concentration of 1:1000, 0.5:1000 and 1:1000, respectively. Secondary antibodies were anti-mouse Cy3 (Amersham Biosciences, Uppsala Sweden) and anti-rabbit Alexa 568 (Molecular Probes, Eugene, United States). Three rounds of washes with 1XPBS 0.1% Tween 20 were carried out. After that, progressive alcohol dehydration was performed, followed by nuclear staining with 4′,6-diamidino-2-phenylindole (DAPI) for fluorescent DNA counterstaining for microscopy. DAPI was added at a final concentration of 2.5 μg/mL in Vectashield Mounting Medium. Fluorescence signals were visualized under an Olympus BX microscope equipped with epifluorescent optics specific for each fluorochrome. Images were captured and analyzed using Cytovision software (Applied Imaging, Inc., Michigan, MI, USA).

### 4.4. γH2AX Immunostaining Combined with a BrdU Assay

HMECs were cultured for 30 min with 50 μM BrdU solution at 37 °C and 5% CO_2_. They were rinsed twice in 1× PBS and permeabilized for 20 min with 1× PBS 0.5% TritonX100. After a 1h blocking step (1× PBS 0.1% Tween 20 2% FCS), rabbit monoclonal anti-γH2AX antibodies diluted in the same blocking solution (1:500) were added. Following an overnight incubation, the cover slides were washed 3 times with 1× PBS 0.1% Tween 20 for 5 min and incubated for 1 h with A488 anti-rabbit monoclonal antibodies (Molecular Probes, Eugene, OR, USA) diluted 1:1000 in blocking buffer. After a second washing step (3 times with 1× PBS 0.1% Tween 20 for 5 min), they were fixed with 4% paraformaldehyde for 10 min. After that, they were soaked for another 10 min in a 0.5M HCl 0.1% TritonX100 solution, followed by another 30 min blocking step. Mouse anti-BrdU (1:100) and rabbit anti-γH2AX (1:500) were added and left to incubate overnight. A third washing step was performed before adding the secondary antibodies: 1:200 Cy3 anti-mouse and 1:500 A488 anti-rabbit diluted in blocking solution. Finally, the last washing step was performed, followed by a short wash with distilled water and a dehydration step in alcohol (1 min 70% ethanol, 1 min 85% ethanol and 1 min 100% ethanol) before mounting the slides with DAPI.

### 4.5. Automated Microscopy and Analysis

A minimum of one-thousand cell nuclei per time point from each sample were evaluated under the automated platform using a 60× objective. The system used was a Spot-counting scan system (Spot AX software, Applied Imaging, Newcastle, UK) consisting of an Olympus BX61 epifluorescence microscope, a CCD camera (with specific filters for DAPI, FITC and Cy3), a motorized x-y stage (BX-UCB, Olympus), a focus motor and computer with the software to store the information concerning the number and combination of signals for each cell. This system automatically scans the preselected area and captures the images under the established capture settings. After that, it classifies every cell “on-the-fly” by the signal combination criteria set up by the user. The settings of the system were maintained from the original FISH analysis [19] with a small variation on the % Spot diameter value (280 instead of 200), and number of planes was fixed to 4 for both probes. The system scans and focuses using DAPI and, after recognizing the presence of a cell, captures the other fluorescent dye signals (Cy3 and FITC). Both are acquired as a z-stack, where each step size was 2.1 μM between planes with a total of 4 planes. The same exposure and camera settings are used to acquire the images for each donor sample. Finally, the system has a review screen, where the images could be reloaded, studied and, if necessary, the user can relocate every cell to verify the automatic classification. The signal combination criteria includes any cell containing 1 or 2 pericentrin signals, from 0 to 10 γH2AX foci with a total number of 20 class groups. All the data is imported into spreadsheets, sorted by number of pericentrin signals and area measurements and analyzed for the number of γH2AX per cell. The software measures the area in pixels, but for storage reasons, it converts pixels into a non-international unit obtained from a mathematical formula expressed as a value of 1000. We refer to this value as nuclear area points.

## 5. Conclusions

In order to study the effects of mammography on HMECs, a highly sensitive method was developed to monitor the chromosome DSBs arising from very low irradiation doses, equivalent to those used in the clinical setting. Our Spot-counting system includes a cell cycle marker, which has enhanced the efficiency of automated methods, as well as improved the accuracy of DNA damage assessment. Furthermore, the additional information that this automated system provides opens up new possibilities in terms of radiation sensitivity assessment, such as using the individual γH2AX foci intensity number or recognizing the cell cycle phase.
